# Genetically Engineered *Escherichia coli* Nissle 1917 Synbiotics Reduce Metabolic Effects Induced by Chronic Consumption of Dietary Fructose

**DOI:** 10.1371/journal.pone.0164860

**Published:** 2016-10-19

**Authors:** Chaudhari Archana Somabhai, Ruma Raghuvanshi, G. Nareshkumar

**Affiliations:** Department of Biochemistry, Faculty of Science, Maharaja Sayajirao University of Baroda, Vadodara-390002, India; University of Basque Country, SPAIN

## Abstract

**Aims:**

To assess protective efficacy of genetically modified *Escherichia coli* Nissle 1917 (*Ec*N) on metabolic effects induced by chronic consumption of dietary fructose.

**Materials and Methods:**

*Ec*N was genetically modified with fructose dehydrogenase (*fdh*) gene for conversion of fructose to 5-keto-D-fructose and mannitol-2-dehydrogenase (*mtl*K) gene for conversion to mannitol, a prebiotic. Charles foster rats weighing 150–200 g were fed with 20% fructose in drinking water for two months. Probiotic treatment of *Ec*N (*pqq*), *Ec*N (*pqq-glf-mtl*K), *Ec*N (*pqq-fdh*) was given once per week 10^9^ cells for two months. Furthermore, blood and liver parameters for oxidative stress, dyslipidemia and hyperglycemia were estimated. Fecal samples were collected to determine the production of short chain fatty acids and pyrroloquinoline quinone (PQQ) production.

**Results:**

*Ec*N (*pqq-glf-mtl*K), *Ec*N (*pqq-fdh*) transformants were confirmed by restriction digestion and functionality was checked by PQQ estimation and HPLC analysis. There was significant increase in body weight, serum glucose, liver injury markers, lipid profile in serum and liver, and decrease in antioxidant enzyme activity in high-fructose-fed rats. However the rats treated with *Ec*N (*pqq-glf-mtl*K) and *Ec*N (*pqq-fdh*) showed significant reduction in lipid peroxidation along with increase in serum and hepatic antioxidant enzyme activities. Restoration of liver injury marker enzymes was also seen. Increase in short chain fatty acids (SCFA) demonstrated the prebiotic effects of mannitol and gluconic acid.

**Conclusions:**

Our study demonstrated the effectiveness of probiotic *Ec*N producing PQQ and fructose metabolizing enzymes against the fructose induced hepatic steatosis suggesting that its potential for use in treating fructose induced metabolic syndrome.

## Introduction

Fructose, present in high fructose corn syrup, is known to induce metabolic syndrome leading to type 2 diabetes mellitus, cardiovascular disease and mortality [1, 2]. Fructose is converted to triglycerides (TG) through de novo lipogenesis when taken in excess amount building up lipids in the liver [3]. These lead to elevated blood lipid levels resulting into inflammation, insulin resistance, increased oxidative stress, and blood glucose levels.

High-fructose fed rats develop clinical features of metabolic syndrome and therefore they are used for assessing beneficial effects of various treatments against metabolic syndrome [4]. In rats fed with high fructose diet, probiotic treatment has been shown to significantly reduce oxidative stress, insulin resistance and lipogenesis [5]. Prebiotic such as short chain fatty acids (SCFA) stimulates fatty acid oxidation, inhibits lipogenesis, and glucose production through inhibition of gluconeogenic gene expression [6, 7]. Mannitol, a known prebiotic, converted to SCFA such as butyrate has been demonstrated to confer protection against the development of colon cancer; in the prevention and treatment of the metabolic syndrome [8, 9]. The combination of prebiotic and probiotic known as “synbiotic” synergistically promotes the growth and survival of existing beneficial bacteria along with the newly added probiotic strains in the colon [10].

Pyrroloquinoline quinone (PQQ) is a powerful antioxidant compound synthesized by many Gram negative bacteria but not by humans and human microbiota [11, 12]. It can induce nerve cells regeneration, enhance mitochondrial functions and reproductive capabilities as well as maintain mitochondrial and neuronal function [13]. Our previous work demonstrated that probiotic *E*. *coli* CFR 16 expressing *Vitreoscilla* haemoglobin (VHb) and secreting PQQ protected against CCl_4_ and dimethyl hydrazine induced damage by reducing liver and colon damage mediated by their antioxidant abilities [14, 15] This probiotic treatment also restored neurotransmitter levels which alter in response to oxidative damage [16]. Additionally, probiotic *Ec*N producing PQQ was found to be more effective than orally given PQQ against alcohol and rotenone induced oxidative stress [17, 18].

Uptake of fructose in *E*. *coli* is mediated by phosphotranferase system (PTS) leading to phosphorylated D-fructose in the cytoplasm [19]. On the other hand, *Zymomonas mobilis* encodes a glucose facilitator protein (GLF) which allows efficient uptake of unphosphorylated D-fructose [20]. We used the genes corresponding to enzymes that catabolize fructose to mannitol and 5-keto-D-fructose in *Ec*N. Heterofermentative Lactic acid bacteria, *Lactobacillus brevis* catalyze the conversion of D-fructose directly to D-mannitol by cytosolic mannitol-2-dehydrogenase (MTLK) [21]. Likewise, *Gluconobacter japonicas* NBRC3260 possess membrane bound Fructose dehydrogenase (FDH; EC 1.1.99.11) which catalyzes the oxidation of D-fructose to 5-keto-D-fructose. FDH is used in the diagnosis and food analysis due to its high substrate specificity to D-fructose [22]. In this study, we genetically modified *E*cN producing PQQ to convert fructose to 5-KF and mannitol in gut by incorporating fructose metabolizing enzymes, FDH and MTLK along with GLF protein, respectively (S1 and S2 Tables). The potential of these modified probiotics, *Ec*N (*pqq-fdh*) and EcN (*pqq-glf-mtlK*) producing PQQ and fructose metabolizing enzymes was investigated for ameliorating high fructose induced metabolic syndrome.

## Material and Methods

### Plasmid, Bacterial strains and culture condition

*Ec*N was obtained from Dr.Ulrich Sonnenborn (Ardeypharm GmbH, Loerfeldstrabe 20, Herdecke, Germany) as a generous gift. All plasmid constructs and bacterial strains used in the present study are summarized in S1 and S2 Tables. Routine DNA manipulations were done in *E*. *coli* DH10B (Invitrogen, USA) using standard molecular biology protocols from Sambrook *et al* [23]. For supplementation to different rat groups, probiotics with different metabolic efficacy were grown overnight in Luria Broth at 37°C, re-inoculated in fresh L.B tubes to achieve final colony forming unit (CFU) of 10^9^ per ml. One ml of fresh culture (CFU of 10^9^/ml) was taken from the tube, centrifuged and washed twice with normal saline before oral administration to rats.

### Construction of pJET with *fdh-pqq* under constitutive *tac* promoter

For the constitutive expression of *fdh* under *tac* promoter, *tac* promoter was obtained by polymerase chain reaction amplification using primers listed in S3 Table. *tac* forward primer contains a modification in *tac* promoter at *lac* repressor binding site and *tac* reverse primer contains a portion of *tac* promoter and complementary region of *fdh*. PCR amplification was done using XT20 polymerase (Thermo Scientific, USA) from plasmid pMALp2. Then, *fdh* gene was amplified using gene specific primers from the genome of *Gluconobacter frauteuri* IFO3260 (S3 Table). The amplicons of size 0.2 kb and 3.7 kb were gel eluted, purified mixed and again amplified using *tac* forward primer and *fdh* reverse primer yielding the final amplicon containing *fdh* under constitutive *tac* promoter (ctac*-fdh) of size 3.9kb. The ctac*-fdh amplicon was gel eluted, purified and ligated in pJET plasmid which gave pAN2. Construct was confirmed by restriction digestion pattern. The activity of the clone was confirmed by fructose dehydrogenase enzyme assay upon transforming in *Ec*N:: *vgb-gfp*. For the constitutive expression of *pqq* operon, *pqq* was amplified by polymerase chain reaction amplification using primers from the genome of *G*. *suboxydans*. This amplicon ctac*-pqq was gel eluted, purified and ligated in pJET plasmid. Further, pAN2 was linearized using *Xho*I and end-filled. Digestion of pAN1 with *Bgl*II gave *pqq* operon which was gel eluted, purified and ligated in *Xho*I digested and gel purified pAN2 which gave pAN7 construct. The construct was confirmed by restriction digestion pattern and PCR amplification. This was followed by transformation of the final construct pAN7 in *Ec*N:: *vgb-gfp*. The construct was confirmed by restriction digestion pattern and PQQ quantification in the bacterial supernatant using fluorometer (Hitachi High-Technologies Corporation, Japan).

### Construction of pJET with *pqq-glf-mtlK* under constitutive *tac* promoter

The strategy for constitutive expression of *mtl*K gene was similar to that of *fdh* gene. The promoter was first amplified by polymerase chain reaction using primers listed in S3 Table. Then, *mtl*K gene was obtained using polymerase chain reaction by primes listed in S3 Table from pRSETmtlK. The amplicon ctac*-mtlK gene was gel eluted, purified and ligated in pJET vector which gave pAN4. The *glf* gene was amplified from *Zymomonas mobilis* using primers listed in S3 Table. The ctac*-glf gene was then gel eluted, purified and ligated in pJET vector which gave pAN3. Digestion of pAN1 by *Bgl*II gave ctac*-pqq gene cluster which was gel eluted, purified and ligated in *Xho*I digested, gel purified pAN3. Construct was confirmed by restriction digestion pattern. Finally, the ctac*-*pqq* and ctac*-*glf* genes were amplified together and the product containing ctac*pqq-ctac*glf was inserted in *Xho*I digested, gel eluted purified vector pAN4 to give pAN6. This was followed by transformation of the final construct pAN6 in *Ec*N:: *vgb-gfp*. Functionality of construct was confirmed by mannitol-2-dehydrogenase enzyme assay. Briefly the cells grown in M9 minimal medium [23] were harvested aseptically at stationary phase by centrifugation at 9,200 g for 2 min at 4°C. Cell-free extract was prepared by sonication [24]. Mannitol-2-dehydrogenase activity was assayed by measuring the rate of oxidation of NAD(P)H using fructose as substrate according to Liu *et al*. [21]. The culture supernatant collected at the end was used for gluconic acid estimation produced by *Ec*N (pAN7) and *Ec*N (pAN6) by HPLC [24].

### PQQ quantification

PQQ was extracted from E.*coli* strains and liver tissue as described in Suzuki *et al*. [25] and Singh *et al*. [17]. Briefly the cells were grown overnight in M9 minimal medium containing glucose. These cells were harvested, and the supernatant was used for PQQ extraction. Culture supernatant was incubated with 50% acetonitrile at 65°C for 2 hours followed by centrifugation at 15,000xg for 10 minutes. The clear supernatant attained was dried using concentrator in vacuum. The residues were dissolved in 50% n-butanol (1 mg/ml) and incubated at 50°C till it dries. Finally, the residues attained were dissolved in water and filtered with 0.2 micron filter. Quantification was done fluorimetrically as described by Suzuki and colleagues (1990) using Hitachi fluorescence spectrophotometer (Hitachi High-Technologies Corporation, Tokyo, Japan) with excitation 375 nm and emission 465 nm (uncorrected). Standard plot for area under curve was drawn using 6 different concentration of standard PQQ ranging from 0.2 to 20 μM. Liver tissue and colonic contents were homogenized in phosphate-buffered saline 20 and 10% (w/v), respectively followed by centrifugation at 10,000 xg for 20 minutes to obtain supernatant for PQQ extraction followed by quantification as described above.

### Animals

Adult albino male Charles Foster rats (180–200 g) were used for animal studies. All rats were housed in plastic cages and maintained in controlled condition as per committee guidelines *i*.*e*. temperature (25 ± 1°C), relative humidity (45.5%) and photoperiod cycle (12 h light: 12 h dark)). Free access to food and water was provided as per recommended by committee for the purpose of control and supervision of experiments on animals (CPCSEA) guidelines of animal ethical committee of M. S. University of Baroda, India, Registration number 938/a/06/CPCSEA. The presented research was approved by Animal Ethical Committee of Department of Biochemistry, The M. S. University of Baroda, Gujarat, India (Approval No. 938/a/06/CPCSEA), and CPCSEA (Committee for the Purpose of Control and Supervision of Experiments on Animals). The rats were acclimated to laboratory conditions, monitored daily twice for welfare without disturbing and checked whether each rat is feeding and drinking. Rats were checked for any red staining around the eyes and physically examined by running fingers over their body to check whether they are normal. Rat body weight and food consumption were measured weekly.

### Experimental design

For present study, rats were divided into seven different groups (*n* = 6) as follows: Control group received pellet diet; Fructose group received pellet diet and 20% fructose in drinking water; F + *Ec*N-2 group received pellet diet, 20% fructose in drinking water and *Ec*N-2; F + *Ec*N (*pqq*) group received pellet diet, 20% fructose in drinking water and *Ec*N (*pqq*); F + *Ec*N (*pqq-glf*) group received pellet diet, 20% fructose in drinking water and *Ec*N (*pqq-glf*); F + *Ec*N (*pqq-glf-mtlK*) group received pellet diet, 20% fructose in drinking water and *Ec*N (*pqq-glf-mtl*K); F + *Ec*N (*pqq-fdh*) group received pellet diet, 20% fructose in drinking water and *Ec*N (*pqq-fdh*). All groups except Control group and Fructose Control group were gavaged with probiotics (10^9^ *cfu*) once per week till two months as per Singh *et al*. [17].

### Colonic SCFA extraction and quantification

Colonic SCFA extraction and quantification was carried out as described in Singh *et al*. [17]. In brief, after dissection of rats colonic content was collected immediately and stored at -80°C till use. For quantification, samples were taken from -80°C and suspended in sterile deionized water containing 0.015 M H_2_SO_4_ and detected using Shimadzu HPLC system with C-18 column. (Shimadzu Analytical (India) Pvt.Ltd, Mumbai, India.

### Preparation of cell lysate and tissue homogenates

Blood was collected by orbital sinus puncture under general anaesthesia with ether in EDTA coated and normal tubes followed by centrifugation at 1500 g for 10 minutes. Plasma and serum were collected separately in fresh tubes and stored in -80°C till use. Pack cell volume (PCV) was washed thrice with normal saline prior to lysis in ice cold water. Cell lysate so obtained was centrifuged at 15,000 g for 10 min and fresh supernatant was used for enzyme assays. Liver was collected and washed with PBS immediately after sacrificing rats by cervical dislocation Liver homogenates were prepared in different buffers for antioxidant enzyme activity.

### Biochemical assays

Superoxide dismutase (SOD) activity was measured by a method which is based on auto-oxidation of pyrogallol monitored spectrophotometrically at 420 nm [26]. Catalase (CAT) activity were monitored by measuring rate of disappearance of hydrogen peroxide (H_2_O_2_) spectrophotometrically at 240 nm [27]. SOD and CAT activities were reported as units/mg protein. Reduced glutathione (GSH) was performed as described in Beutler *et al*. [28]. Lipid peroxidation was estimated by measuring levels of malondialdehyde at 412 nm as described in Buege *et al*. [29].

### Liver enzyme test, kidney function test, and lipid estimation

Aspartate transaminase(AST), Alkaline phosphatase(ALP), and Alanine transaminase(ALT),Total bilirubin, triglyceride, HDL, VLDL and total cholesterol content in blood plasma were measured using kits as per manufacturer protocol (Beacon Diagnostics Pvt. Ltd. Navsari, India).

### mRNA expression and qRT-PCR

RNA was extracted with Trizol (Invitrogen Bio Services India Pvt. Ltd., Bangalore, India) and cDNAs were generated from 1 μg total RNA (Reverse Transcription Kit; Applied Bio systems, Foster City, CA) following the manufacturer’s instructions. The primers for fatty acid synthase and acyl coenzyme A gene were ACCTCATCACTAGAAGCCACCAG (forward) and GTGG*TAC*TTGGCCTTGGGTTTA (reverse), and CCCAAGACCCAAGAGTTCATTC (forward) and TCACGGATAGGGACAACAAAGG (reverse), respectively. PCR was performed using ABI Quant-StudioTM 12K flex Real Time PCR system coupled with SYBR Green technology (Applied Biosystems) and following cycling parameters. The linearity of the dissociation curve was analysed using the software provided with the thermo cycler (QuantStudioTM). Each sample was analysed in duplicate. The mean cycle time of the linear part of the curve was designated Ct.

### Histopathological changes

Liver tissue was fixed in 10% neutral buffered formalin. Histological sections were stained with hematoxylin and eosin and evaluated by pathologist unaware with experimental codes.

### Statistical analysis

All values are expressed as mean ± SEM. Differences in lipid peroxidation and antioxidant enzymes (SOD, CAT and GSH) among six different groups were evaluated using the one-way ANOVA followed by Bonferroni comparisons. Differences were considered significant at P<0.05. Calculations were performed using commercially available statistical software packages (Graph Pad PRISM Version 5.0 La Jolla, CA 92037 USA).

## Results

### Cloning and expression of *pqq-glf-mtl*K and *pqq-fdh* in *Ec*N::*vgb-gfp*

The pAN1 plasmid possessing *G*. *suboxydans pqq* operon, the pAN2 plasmid containing *G*. *frauteuri* IFO3260 *fdh* gene cluster, the pAN3 plasmid harbouring *Z*. *mobilis glf* gene, the pAN4 plasmid containing *L*. *brevis mtlK* gene, the pAN5 plasmid possessing *pqq* operon and *glf* gene together, the pAN6 plasmid containing *pqq* operon, *glf* gene and *mtlK* together, the pAN7 plasmid harbouring *pqq* operon and *fdh* together under constitutive *tac* promoter were cloned in pJET vector (S1 Fig). Functional confirmation of *Ec*N transformants harbouring pAN1, pAN5, and pAN6 and pAN7 plasmids was done by growth and acidification on Tris-buffered medium containing methyl red as pH indicator [30].

In M9 medium, *Ec*N containing pAN1, pAN5, pAN6 and pAN7 produced 6.5 ± 0.15 μg / ml, 6.29 ± 0.22 μg / ml, 5.78 ± 0.18 μg / ml and 5.23 ± 0.32μg / ml of PQQ/ml along with 34.44± 0.09 mM, 31.44± 0.17 mM, 30.56± 0.24 mM and 31.34 ± 0.19 mM of gluconic acid in the culture medium. Acidification of Tris-buffered medium with methyl red as indicator is due to secretion of gluconic acid by PQQ dependent glucose dehydrogenase (Data not provided). In M9 medium, *Ec*N harbouring *mtlK* and *pqq-glf-mtlK* showed 0.23± 0.22 and 0.2± 0.16 μmoles of NADPH oxidized per min per mg of protein enzyme activity.

### Effect of probiotic treatment on body weight, hyperglycemia

The body weight gain of fructose fed group significantly increased compared to control group. After administration of probiotic producing fructose metabolizing enzymes, *Ec*N (*pqq-glf-mtlK*) and *Ec*N (*pqq-fdh*) body weight gain significantly decreased and reached the levels similar to the control group (Table 1). The fasting serum concentrations of glucose were also significantly higher in fructose fed group compared to control group. However, the increase in serum concentrations of glucose, in fructose fed rats were significantly decreased after administration of probiotic producing fructose metabolizing enzymes (Table 1).

**Table 1:** Effect of different probiotic treatment on body weight gain, food intake, fasting glucose, serum insulin levels of rats.

Groups	Body weight gain (g)	Food intake (g/day)	Fasting glucose (mg/dl)	Serum insulin (μg/l)
**Control (No F)**	43.33±8.45	18.15±1.19	98.04±3.59	1.10±0.21
**Fructose control (F)**	92.6±8.902**	17.24±1.73	188.48±6.83***	3.14 ± 0.25**
**F+ *Ec*N-2**	87±9.08**	17.66±1.14	185.57±4.81***	3.28± 0.35**
**F+ *Ec*N (*pqq*)**	67.75±9.12**	17.77±1.25	154.33±5.34***	2.91± 0.15**
**F+ *Ec*N (*pqq-glf*)**	66.75±9.23**	17.69±1.58	149.82±10.31***	2.88 ± 0.21**
**F+ *Ec*N (*pqq-glf-mtl*K)**	52.4±7.31^**# #**^	17.33±1.93	105.23±10.49**^# #^	1.23± 0.35^# #^
**F+ *Ec*N (*pqq-fdh*)**	60.75±8.93^**# #**^	17.97±1.25	120.29±6.50**^# #^	1.57± 0.35^# #^

F, Fructose.

Values are expressed as mean ± SEM (*n* = 6 each group).

** P ≤ 0.01 and

*** P ≤ 0.001 compared with control group.

# # P ≤ 0.01 compared with fructose control group.

### Effect of probiotic treatment on hepatic injury markers

**Fig 1:**
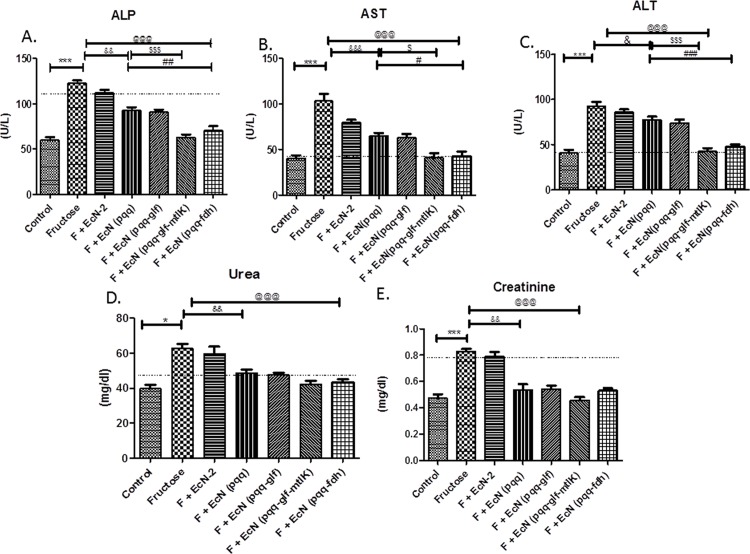
Effect of probiotic treatment on liver enzyme tests. (A) ALP (B) AST (C) ALT activity and kidney function tests (D) Urea (E) Creatinine of serum. Values are expressed as mean ± SEM (n = 5–6 each group). ***P≤ 0.001 compared with fructose control, ^@@@^P ≤ 0.001 compared to fructose control, ^$ $ $^ P ≤ 0.001, ^$ $^ P ≤ 0.01 compared to EcN (*pqq*) group. F: Fructose.

Increased serum levels of Aspartate transaminase (AST), Alkaline phosphatase (ALP), and Alanine transaminase (ALT) were found in fructose fed group (Fig 1A–1C). These results showed that fructose induced damage to liver cells. However, supplementation of probiotic producing fructose metabolizing enzymes significantly decreased ALP, AST and ALT levels in fructose fed group.

### Effect of probiotic treatment on kidney function

There is a noticeable increase in urea and creatinine in kidneys of rats fed with fructose fed group compared to control group. Treatment with probiotic producing fructose metabolizing enzymes, *Ec*N(*pqq-glf-mtlK*) and *Ec*N(*pqq-fdh*) decreased urea and creatinine in probiotic treated rats as compared to fructose fed group (Fig 1D and 1E).

### Effect of probiotic treatment on dyslipidemia

**Fig 2:**
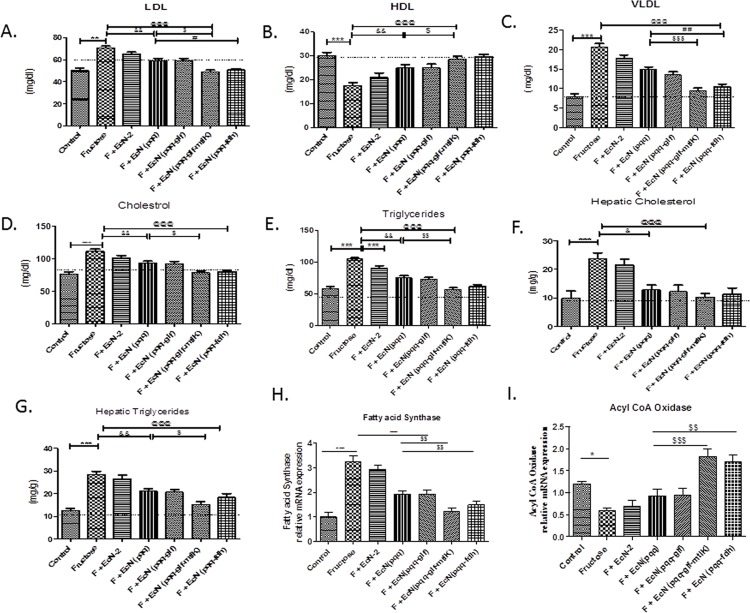
Effect of probiotic treatment on serum lipid profile. (A) LDL, (B) HDL, (C) VLDL, (D) Cholesterol and (E) Triglycerides; on hepatic lipid profile (F) Cholesterol and (G) Triglycerides; (H) mRNA of Fatty acid synthase and (I) mRNA of Acyl Coenzyme A oxidase. Values are expressed as mean ± SEM (n = 5–6 each group). ***P≤ 0.001 compared with fructose control, ^@@@^P ≤ 0.001 compared to fructose control, ^$ $ $^ P ≤ 0.001, ^$ $^ P ≤ 0.01 compared to *Ec*N (*pqq*) group, ^###^ ≤ 0.001. Values are expressed in mg/dl. F: Fructose.

Higher serum levels of LDL, VLDL, cholesterol and TG were noticed in fructose fed group as compared to control group (Fig 2A, 2C, 2D and 2E). Administration of probiotic producing fructose metabolizing enzymes, significantly reduced the increased levels of LDL, TG, VLDL and cholesterol as compared to fructose fed group. Our results demonstrated that higher levels of hepatic TG and cholesterol were found in fructose fed group as compared to control group. Feeding of probiotic producing fructose metabolizing enzymes significantly suppressed the increased hepatic TG and cholesterol levels compared to fructose fed group which showed that probiotic can suppress the formation of hepatic steatosis (Fig 2F and 2G).

### Effect of probiotic treatment on Hepatic Fatty Acid Synthase (FAS) and Acyl Coenzyme A Oxidase (ACOx) mRNA Expression

Finally, mRNA expression of FAS and ACOx showed increased and decreased expression, respectively, in fructose fed group (Fig 2H and 2I). This expression was reversed in groups supplemented with probiotic producing fructose metabolizing enzymes.

### Effect of probiotic treatment on oxidative stress

**Fig 3:**
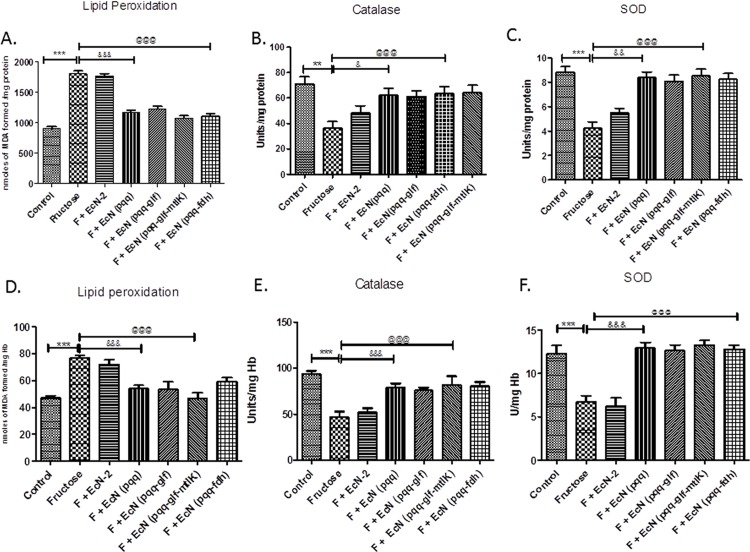
**Effect of probiotic treatment on antioxidant status of liver (A), (B) and (C) and blood (D), (E) and (F) in rats.** (A and D) Lipid peroxidation (LPO), (B and E) Catalase and (C and F) SOD. Values are expressed as mean ± SEM (n = 5–6 each group). ***P≤ 0.001 compared with fructose control, ^@@@^P ≤ 0.001 compared to fructose control, $ $ $ P ≤ 0.001, ^$ $^ P ≤ 0.01 compared to *Ec*N (*pqq*) group. F: Fructose.

Fructose control group rats showed markedly high levels of lipid peroxidation as compared to control rats (Fig 3A) After administration of probiotic producing fructose metabolizing enzymes, the levels of MDA were significantly lowered to near normal levels as compared to untreated fructose fed rats group. Catalase and SOD activities in rat livers were decreased in fructose fed group, as compared to control group (Fig 3B and 3C). Probiotic treatment significantly up regulated these two antioxidant enzyme activities, which were decreased in fructose fed group, in rat liver. Glutathione levels decreased in fructose fed rats compared to control group (Table 2). Treatment with probiotic producing fructose metabolizing enzymes increased glutathione levels as compared to fructose fed rats.

**Table 2:** Effect of probiotic treatment on Colonic SCFA, PQQ concentration in liver tissue and fecal samples and liver GSH levels in rats.

Groups	SCFA levels			PQQ levels		Liver GSH(μmoles/g tissue)
	Acetate	Propionate	Butyrate	Feacal (n moles /g faecal wet weight)	Liver (picomoles / g tissue)	
**Control (No F)**	98.43±3.28	18.52±4.24	9.52±1.41	0.698±0.10	30.93±1.13	22.41±1.15
**Fructose control (F)**	80.32±6.94*	20.54±3.67	11.10±1.41	0.656±0.09	34.23±2.34	11.66±1.24**
**F+ *Ec*N-2**	76.45 ±4.89	25.53 ± 5.11	10.99±2.91	0.701±0.11	39.23±1.54	14.9 ±3.1**
**F+ *Ec*N (*pqq*)**	123.12±7.61^##^	28.16±3.65^#^	18.54±2.56^#^	4.181±0.13**	58.24±4.08**	17.54 ±2.06**
**F+ *Ec*N (*pqq-glf*)**	120.32 ±4.5^##^	29.95618^#^	19.24±4.34^#^	3.633±0.04**	57.89±.94**	16.94±1.75**
**F+ *Ec*N (*pqq-glf-mtl*K)**	125.27±3.45^##^	30.57512^#^	20.56±3.4^#^	3.51± 0.09**	60.32±1.97**	19.23±1.86^##^
**F+ *Ec*N (*pqq-fdh*)**	126.12±6.61^##^	28.96±3.65^#^	19.84±3.9^#^	3.69± 0.09**	52.43±0.97**	18.93±1.66^##^

F, Fructose; SCFA, Short chain fatty acid; GSH, Reduced glutathione; PQQ, pyrroloquinoline quinone.

Values are expressed as mean ± SEM (*n* = 6 each group).

*P ≤ 0.05 and

** P ≤ 0.01 compared with control group.

^#^ P ≤ 0.05 and

^##^P ≤ 0.001 compared with Fructose control group.

### Effect of probiotic treatment on colonic SCFAs concentration

Additionally, levels of colonic SCFA *i*.*e*. acetate, propionate and butyrate were found to be increased in EcN (*pqq*), EcN (*pqq-glf*), EcN (*pqq-glf-mtlK*) and EcN (*pqq-fdh*) fed rats in comparison with control rats and fructose fed rats (Table 2).

### Effect of probiotic treatment on PQQ concentration in faecal matter and liver

PQQ concentration estimated in faecal matter and in liver of *Ec*N (*pqq*), *Ec*N (*pqq-glf*), *Ec*N (*pqq-glf-mtlK*) and *Ec*N (*pqq-fdh*) co-treated rats, on the day of sacrifice, was found to be increased than control and fructose-treated rats (Table 2). Rats treated with *Ec*N-2 and fructose fed rats had similar PQQ concentration.

### Histopathological analysis

High degree of lipid droplet accumulation was found in Group 2 rats while Group 6 and 7 rats, supplemented with probiotic producing fructose metabolizing enzymes, showed significant reduction in lipid droplet accumulation which was correlated with decreased serum and hepatic triglyceride levels (S2 Fig).

## Discussion

Fructose which is highly lipogenic has now become a major constituent of our modern diet even though it was absent in our diet few hundred years ago [31]. In several studies it has been observed that acute fructose ingestion contributes to the synthesis of hepatic triose-phosphate leading to fatty acid synthesis [32].

S3 and S4 Figs. show the proposed mechanism of conversion of fructose by probiotic producing fructose metabolizing enzymes, *Ec*N (*pqq-glf-mtlK*) and *Ec*N (*pqq-fdh*). Fructose is taken up by probiotic *Ec*N (*pqq-glf-mtlK*) through GLF in unphosphorylated form whereby the cytosolic MTLK then converts it to mannitol which is exported outside the *Ec*N (*pqq-glf-mtl*K). The membrane bound fructose dehydrogenase converts the fructose to 5-KF which is then exported outside the EcN (*pqq-fdh*).

Fructose is well known for inducing metabolic syndrome. 20% fructose increased fasting glucose similar to the report of Mamikutty *et al*. [33]. The efficiency of probiotics in ameliorating metabolic disorders has been revealed in a high-fructose-fed rat model [14]. Probiotic producing fructose metabolizing enzymes treatment maintained body weight and fasting glucose level in comparison with fructose control group. In our present data, the serum levels of two critical markers of liver injury, ALT and AST, were increased in fructose control rats. The activities of hepatic antioxidant enzymes, SOD and catalase were decreased in fructose control rats. These results demonstrated that high fructose led to production of enhanced oxidative stress in rats, which then resulted in liver damage in fructose fed rats seen in our study. After treatment with probiotic, up regulation of hepatic activities of antioxidant enzymes and down regulation of serum AST and ALT levels in high fructose rats were noticed. These findings implied that consumption of probiotic significantly reduced both liver damage and oxidative stress in fructose fed rats by enhancing hepatic antioxidants expressions and uptake and conversion of fructose in the intestine by probiotic.

High fructose increased plasma TGs, most probably by up regulation of hepatic de novo lipogenesis and secretion of TGs [34]. Administration of probiotic producing fructose metabolizing enzymes significantly reduced the levels of important components of metabolic disorder, including LDL, TG, and cholesterol, enhanced by fructose. In addition to serum levels, the increased hepatic lipid accumulation by fructose was also found to be suppressed by oral administration of probiotic producing fructose metabolizing enzymes. The levels of SCFA increased in probiotic treated group suggest that fructose is converted to mannitol which is further converted to SCFA in intestine by colonic flora. This is supported by the fact that mannitol treatment increased levels of butyrate in large intestine of pigs [12]. Presence of PQQ enables glucose dehydrogenase to produce gluconic acid which in turn is utilized by *Bifidobacteria* and *Lactobacilli* species leading to the formation of SCFA [35].

*Ec*N is known to have many probiotic properties, acts as a safe carrier for localized delivery of biomolecules in intestine for human use [36]. S5 Fig shows the proposed mechanism of action of probiotic producing PQQ and fructose metabolizing enzyme, *Ec*N (*pqq-glf-mtlK*). *Ec*N possessing MTLK converts fructose to mannitol while secreted PQQ acts as an antioxidant molecule as well as a co-factor for glucose dehydrogenase enzyme which catalyzes the production of gluconic acid. Thus, *Ec*N (*pqq-glf-mtlK*) facilitates the formation of two prebiotic molecules, mannitol and gluconic acid. Mannitol and gluconic acid are metabolized by lactic acid bacteria in lower part of gastrointestinal tract resulting in production of SCFA [12, 35]. Vgb produced by *Ec*N could improve the survival in intestine as seen in probiotic *E*. *coli* CFR16 strain [14]. Overall metabolic effects observed in the present study demonstrates the synergistic effects of *Ec*N, PQQ, Vgb and SCFA formed due to in situ generation of prebiotic gluconic acid and mannitol. Thus, these *Ec*N probiotics act as synbiotics in the intestine.

Recent studies have recommended that the incorporation of a synbiotic with antioxidants can help in alleviating certain disease states *via* synergistically improved intestinal microflora [37, 38, 39]. Use of genetically modified probiotics is more beneficial than wild-type probiotics by not only acting as a suitable vehicle for the delivery of small molecules but also colonize more efficiently in the gut as most probiotic bacteria do not colonize properly in unhealthy individuals [40]. This is supported by the fact that *Ec*N secreting PQQ was very efficient in preventing adverse effects of chronic ethanol consumption [18]. Thus, this approach may be very useful in developing novel synbiotics for treatment of chronic medical conditions including obesity.

Our study supports the concept of sustained delivery of molecules for treatment of fructose induced hepatic steatosis. This will decrease the need for daily administration of molecules whereby it can be effective strategy in the treatment of metabolic syndrome.

## Supporting Information

S1 Fig Agarose gel analysis of PCR amplicons of the recombinant plasmids (A) pAN1, (B) pAN5, (C) pAN6 and (D) pAN7.
(TIF)

S2 Fig Haematoxylin and Eosin staining of rat liver tissue. Blue arrows indicate the accumulation of lipid droplets in Hepatocytes of fructose fed rat (Fructose control). Images were taken by LEICA DME microscope at 40 X magnification (A) Control, (B) Fructose control, (C) EcN-2, (D) EcN (*pqq-glf-mtlK*) and (E) EcN (*pqq-fdh*).
(TIF)

S3 Fig Proposed mechanism of fructose conversion to mannitol by probiotic *Ec*N (*pqq-glf-mtlK*). PTS: Phosphotransferase system, MtlK: Mannitol dehydrogenase.
(TIF)

S4 Fig Proposed mechanism of fructose conversion to 5-KF by probiotic *Ec*N (*pqq-fdh*). 5-KF: 5-Ketofructose, FDH: Fructose dehydrogenase.
(TIF)

S5 Fig Proposed mechanism of probiotic *Ec*N (*pqq-glf-mtlK*) producing PQQ and mannitol dehydrogenase in gut on fructose induced metabolic effects. (TIF)

S1 Table Plasmids used in this study. Ap = Ampicillin, r = resistance. (DOCX)

S2 Table Bacterial strains used in this study. (DOCX)

S3 Table List of primers used in this study. ^a^Primers used for construction of ptac*-pqq-fdh.^b^Primers used for construction of ptac*-pqq—glf-mtlK.
(DOCX)

## Abbreviations

*Ec*N: *Escherichia coli* Nissle 1917
TG: Triglycerides
Fdh: Fructose dehydrogenase
MTLK: mannitol-2-dehydrogenase
PQQ: pyrroloquinoline quinone
SCFA: short chain fatty acids
GLF: glucose facilitator protein
CFU: colony forming unit
SOD: Superoxide dismutase
PCV: Pack cell volume
CAT: Catalase
GSH: glutathione
AST: Aspartate transaminase
ALP: Alkaline phosphatase
ALT: Alanine transaminase
vgb: *Vitreoscilla* haemoglobin
H_2_O_2_: Hydrogen peroxide
MDA: Malondialdehyde
