# Supplementary figures and images for: Genetically Engineered Escherichia coli Nissle 1917 Synbiotics Reduce Metabolic Effects Induced by Chronic Consumption of Dietary Fructose
Source: PLoS One. 2016 Oct 19;11(10):e0164860. doi: 10.1371/journal.pone.0164860 (PMC5070853; doi:10.1371/journal.pone.0164860)

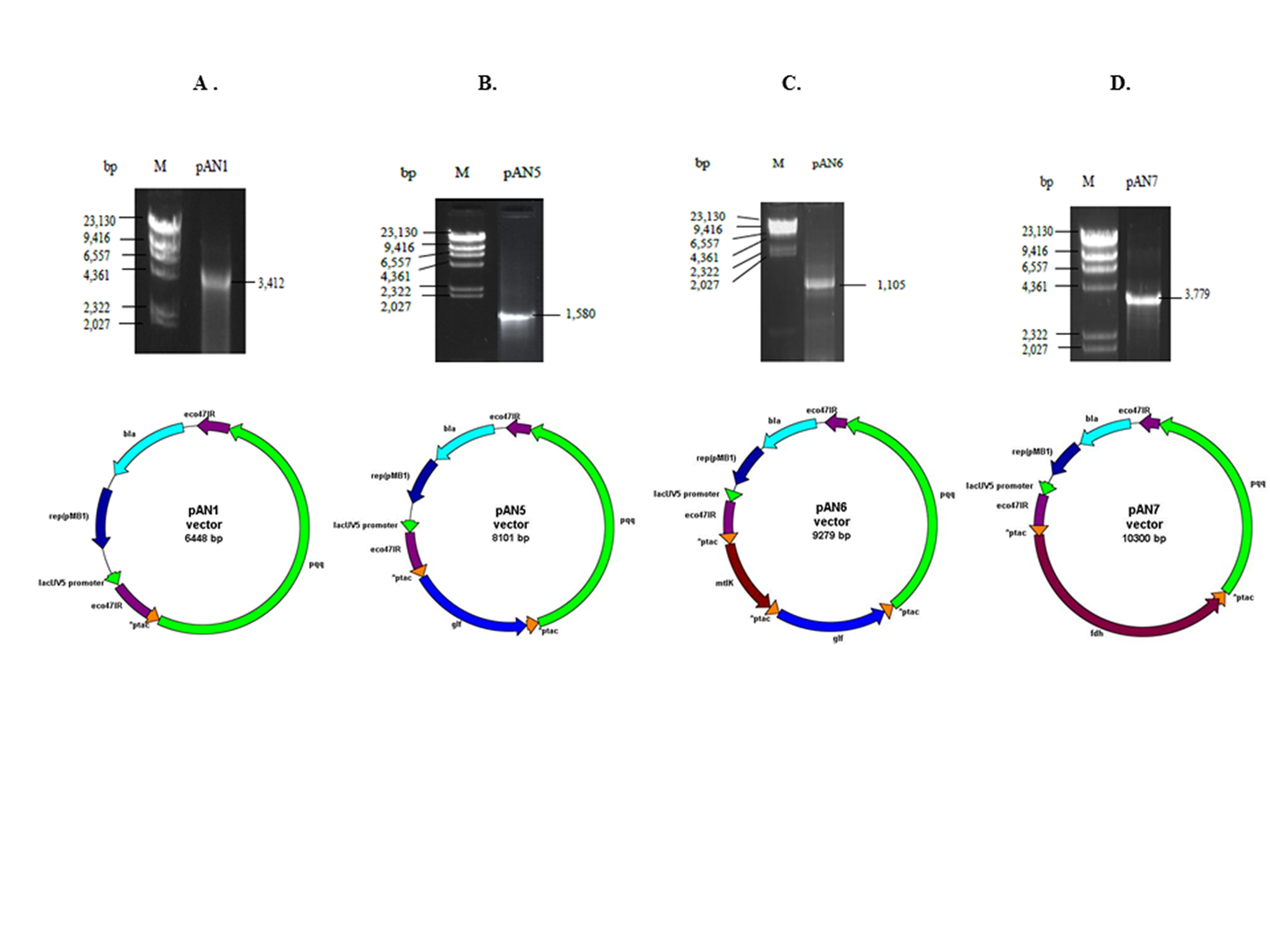

Supplement: S1 Fig — Agarose gel analysis of PCR amplicons of the recombinant plasmids (A) pAN1, (B) pAN5, (C) pAN6 and (D) pAN7. (TIF) [file pone.0164860.s001.tif]

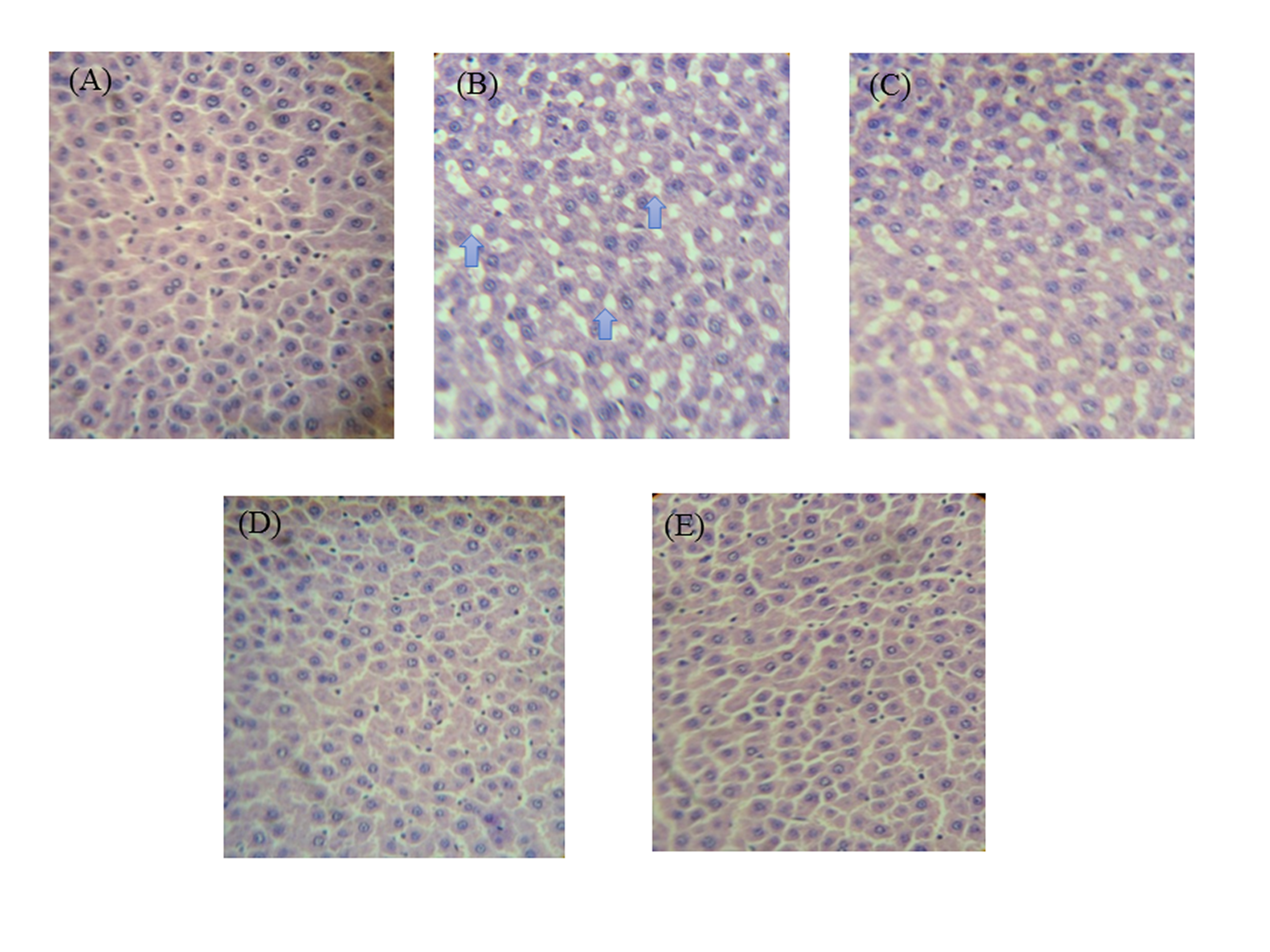

Supplement: S2 Fig — Blue arrows indicate the accumulation of lipid droplets in Hepatocytes of fructose fed rat (Fructose control). Images were taken by LEICA DME microscope at 40 X magnification (A) Control, (B) Fructose control, (C) EcN-2, (D) EcN (pqq-glf-mtlK) and (E) EcN (pqq-fdh). (TIF) [file pone.0164860.s002.tif]

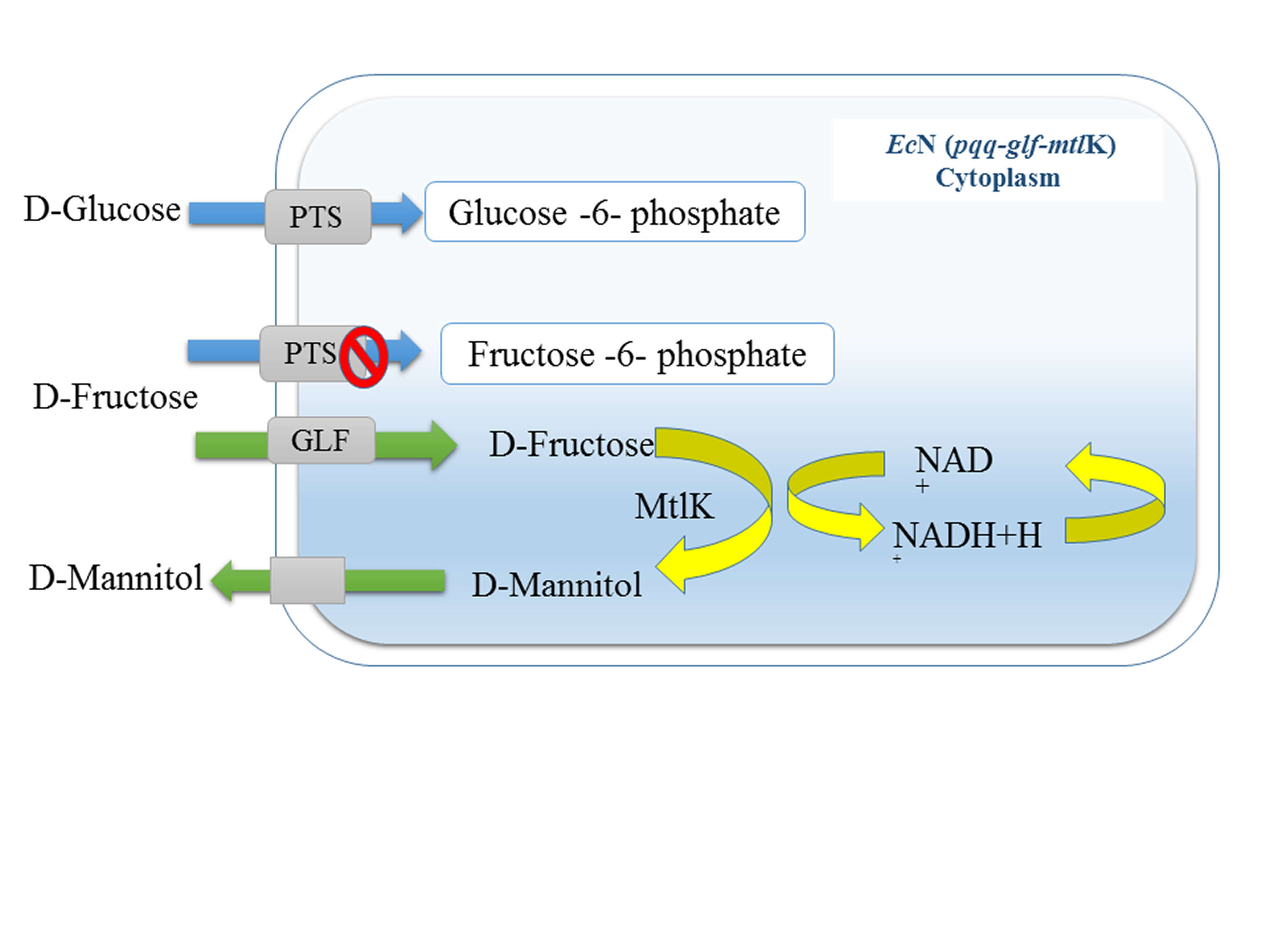

Supplement: S3 Fig — PTS: Phosphotransferase system, MtlK: Mannitol dehydrogenase. (TIF) [file pone.0164860.s003.tif]

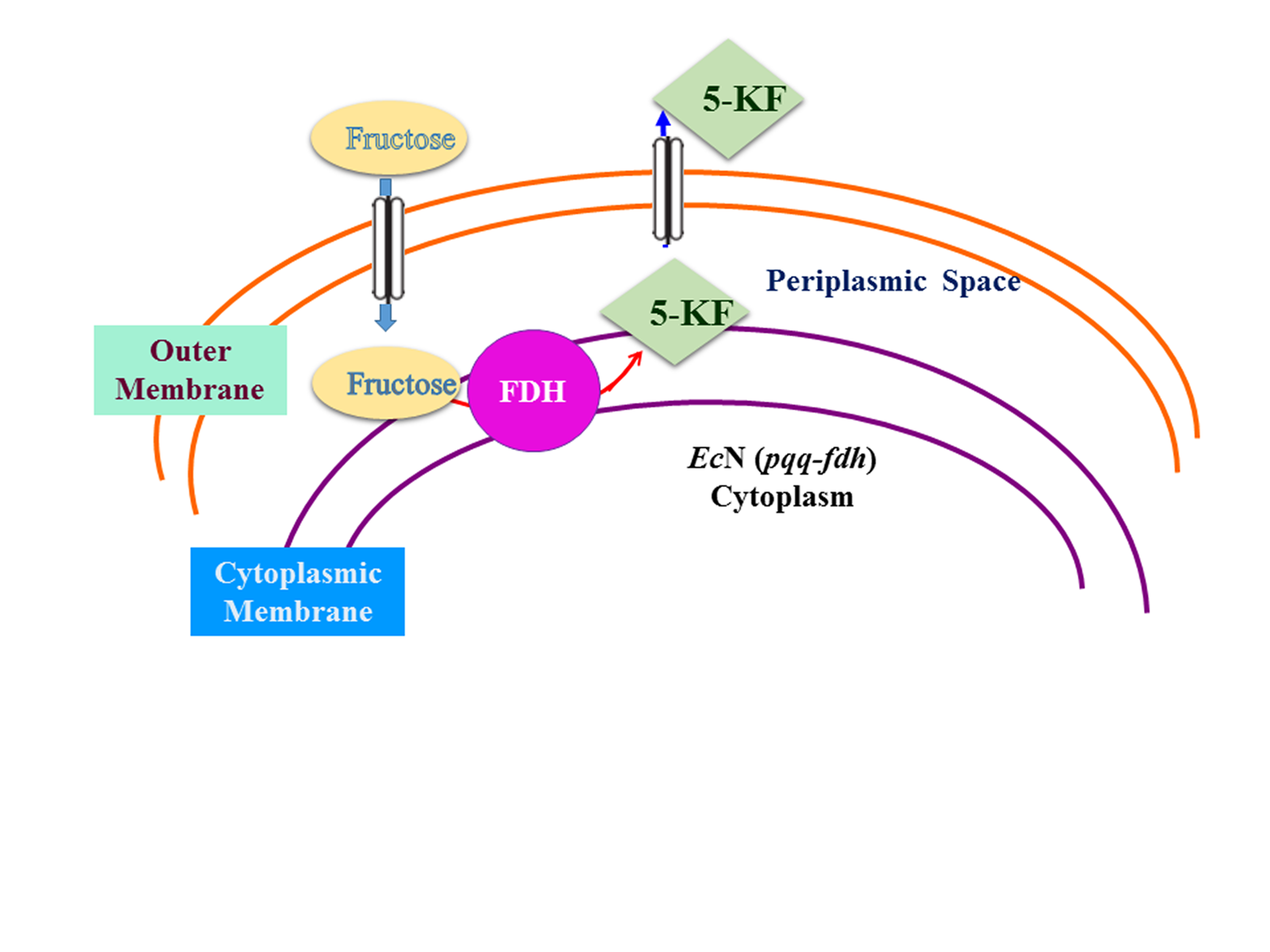

Supplement: S4 Fig — 5-KF: 5-Ketofructose, FDH: Fructose dehydrogenase. (TIF) [file pone.0164860.s004.tif]

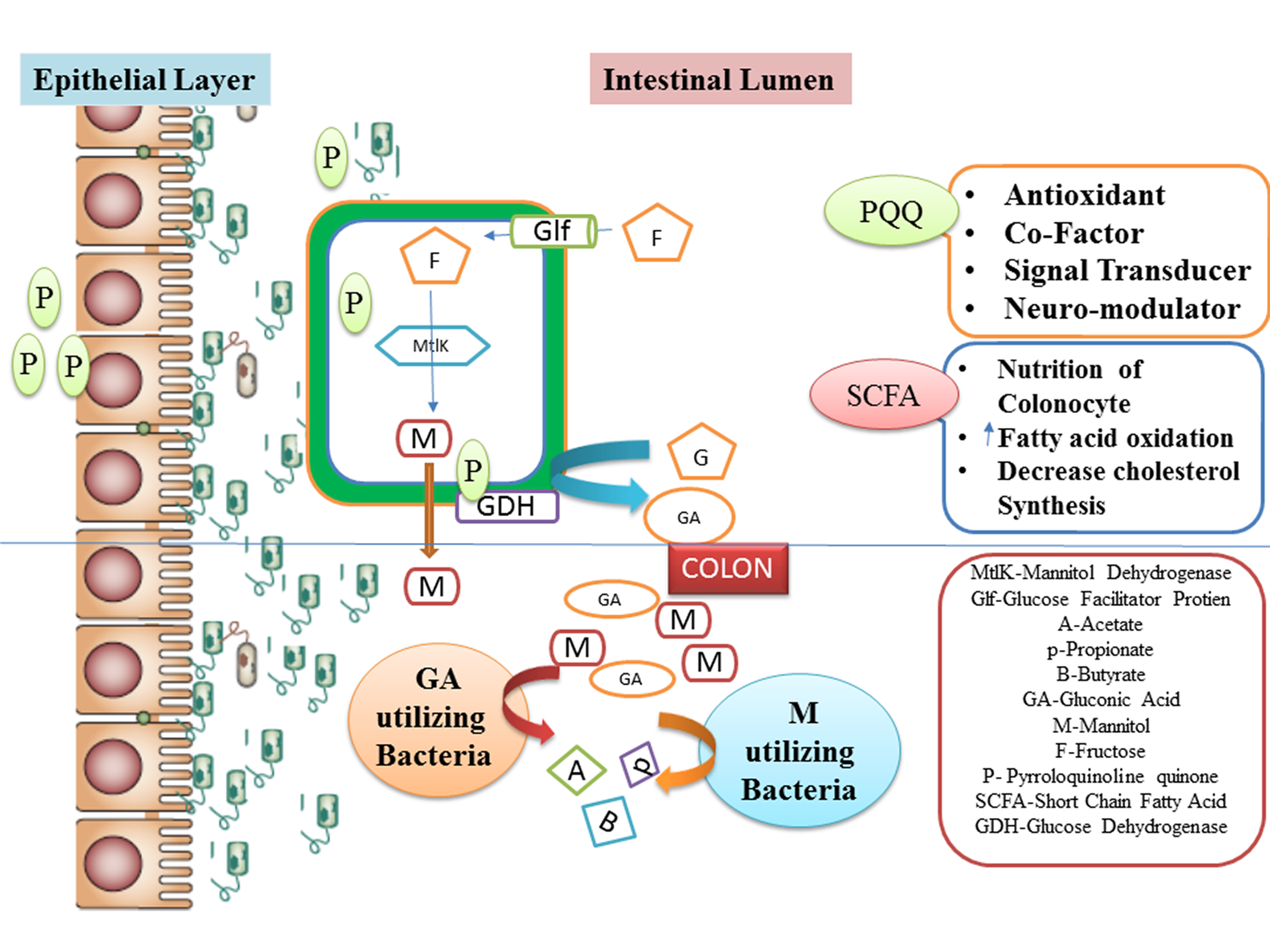

Supplement: S5 Fig — (TIF) [file pone.0164860.s005.tif]
