# Supplementary material for: Genetically Engineered Escherichia coli Nissle 1917 Synbiotics Reduce Metabolic Effects Induced by Chronic Consumption of Dietary Fructose
Source: PLoS One. 2016 Oct 19;11(10):e0164860. doi: 10.1371/journal.pone.0164860 (PMC5070853; doi:10.1371/journal.pone.0164860)
Supplement: S1 Table — (DOCX) [file pone.0164860.s006.docx]

**S1 Table: Plasmids used in this study.**

| Plasmids | Characteristics | Description | Reference |
| --- | --- | --- | --- |
| pJET 2.1 | *placUV5*, rep (pMB1), T7 promoter, Ap^r^ | Blunt end PCR cloning vector | Thermo Scientific CloneJET PCR Cloning Kit |
| pMALp2 | p*tac, malE,*  Ap^r^ |  | NEB |
| (pAN1) | pJET 2.1 with *ptac*-pqq,* Ap^r^ | pJET 2.1 containing *pqq* operon under constitutive *tac* promoter. | This study |
| (pAN2) | pJET 2.1 with *ptac*-fdh,* Ap^r^ | pJET 2.1 containing *fdh* gene under constitutive *tac* promoter. | This study |
| (pAN3) | pJET 2.1 with *ptac*-glf*, Ap^r^ | pJET 2.1 containing *glf* gene under constitutive *tac* promoter. | This study |
| (pAN4) | pJET 2.1 with  *ptac*-mtlK*, Ap^r^ | pJET 2.1 containing *mtl*K gene under constitutive *tac* promoter. | This study |
| (pAN5) | pJET 2.1 with *ptac*-pqq-glf,* Ap^r^ | pJET 2.1 containing *pqq* operon and *glf* genes under constitutive *tac* promoter. | This study |
| (pAN6) | pJET 2.1 with *ptac*-pqq-glf-mtlK*, Ap^r^ | pJET 2.1 containing *pqq* operon, *glf* and *mtlK* genes under constitutive *tac* promoter. | This study |
| (pAN7) | pJET 2.1 with *ptac*-pqq-fdh,* Ap^r^ | pJET 2.1 containing pqq operon and *fdh* genes under constitutive *tac* promoter. | This study |

Ap, Ampicillin; r, resistance.
