# Supplementary material for: Genetically Engineered Escherichia coli Nissle 1917 Synbiotics Reduce Metabolic Effects Induced by Chronic Consumption of Dietary Fructose
Source: PLoS One. 2016 Oct 19;11(10):e0164860. doi: 10.1371/journal.pone.0164860 (PMC5070853; doi:10.1371/journal.pone.0164860)
Supplement: S2 Table — (DOCX) [file pone.0164860.s007.docx]

**S2 Table: Bacterial strains used in this study.**

| Strains | Characteristics | Reference |
| --- | --- | --- |
| *E. coli* DH10B | Host strain for routine DNA manipulation. | (Invitrogen, USA) |
| *Ec*N-2 | *Ec*N containing *vgb* and *gfp* genes in the genome. | Singh *et al.*,2014 |
| *Ec*N(*pqq*) | *Ec*N-2 containing pAN1. | This study. |
| *Ec*N(*pqq-glf*) | *Ec*N*-*2 containing pAN5*.* | This study |
| *Ec*N (*pqq-glf-mtl*K) | *Ec*N*-*2 containing pAN6. | This study |
| *Ec*N (*pqq-fdh*) | *Ec*N-2 containing pAN7. | This study |
