# Supplementary material for: Genetically Engineered Escherichia coli Nissle 1917 Synbiotics Reduce Metabolic Effects Induced by Chronic Consumption of Dietary Fructose
Source: PLoS One. 2016 Oct 19;11(10):e0164860. doi: 10.1371/journal.pone.0164860 (PMC5070853; doi:10.1371/journal.pone.0164860)
Supplement: S3 Table — aPrimers used for construction of ptac*-pqq-fdh.bPrimers used for construction of ptac*-pqq—glf-mtlK. (DOCX) [file pone.0164860.s008.docx]

**S3 Table: List of primers used in this study.**

| **Primer** | **Sequence (5’ – 3’)** |
| --- | --- |
| *tac* forward primer^a,b^ | CGGAATTCCGTTGACAATTAATCATCGGCTCGTATAATGGATCG AATTGTGAGCGGAATCGATTTTCACACAG |
| *tac* reverse primer ^a,b^ | TGTCGTGACATCTTGCATCTCAAATTGTTTTGTACCCGTTAAAACTAAAGCTTTCAT AAT CTA TGG TCC TTG TTG GTG AAG TG |
| *pqq* forward primer ^a,b^ | CAAGGACCATAGATT ATG GCC TGG AAC ACA CCG A |
| *pqq* reverse primer ^a,b^ | CC CTCGAG GG TTA CGT ATA ACG CCT GTA GAA CAA CGT GC |
| *glf* forward primer ^b^ | CGAGCTC G TTGACAATTA ATC ATC GGC TCGTATAATG GATCG AATTGT GAG |
| *glf* reverse primer ^b^ | GACTACTTTCAGAACTCAT AAT CTA TGG TCC TTG TTG GTG AAG TG |
| *mtlK* forward primer ^b^ | CAA GGA CCA TAG ATTATG AAA GCT TTAGTT TTAACG GGTACAAAA CAA TTT GAG ATG CAA GAT GTC ACG ACA CCA ACT GTC |
| *mtlK* reverse primer ^b^ | CG GAATTC CG TTC ACA ACT GCC TTG CTG ACA TGT TCC AC |
| *fdh* forward primer ^a^ | CAA GGA CCA TAG ATT GAGAAGGTA AATG GAAAAAATAGCTGATTC |
| *fdh* reverse primer ^a^ | CG GAATTC CG TTA CCC CTG TTT CAG GTC ATT GAG |

^a^Primers used for construction of *ptac*-pqq-fdh.*

^b^Primers used for construction of *ptac*-pqq--glf-mtlK*.
